# Cholinergic Signaling Modulates Intestinal Pathophysiology in a *Drosophila* Model of Cystic Fibrosis

**DOI:** 10.1101/2025.07.02.662792

**Published:** 2025-07-05

**Authors:** Elizabeth Lane, Afroditi Petsakou, Ying Liu, Weihang Chen, Mujeeb Qadiri, Yanhui Hu, Norbert Perrimon

**Affiliations:** 1 Department of Genetics, Blavatnik Institute, Harvard Medical School, Boston, MA, 7 02115, USA; 2 HHMI, Harvard Medical School, Boston, MA, 02115, USA

## Abstract

Cystic Fibrosis (CF) is a monogenic genetic disease caused by mutations in the Cystic Fibrosis Transmembrane conductance Regulator (CFTR) chloride/bicarbonate channel, which is expressed in certain epithelia cells. Current therapies focus on restoring CFTR function but many gut-related pathologies persist, highlighting the need for complementary treatments to improve the quality of life of patients living with CF. In this study, we use *Drosophila melanogaster* as a model to investigate the gut-specific effects of *Cftr* loss. We demonstrate that enterocyte-specific knockdown of *Cftr* in flies recapitulates several CF pathologies, including reduced intestinal motility, nutrient malabsorption, and decreased energy stores. Using single-nuclei RNA sequencing (snRNA-seq), we identify significant transcriptional changes in the CF model gut, including the upregulation of *acetylcholine esterase (Ace, human AChE),* which leads to reduced cholinergic signaling. Cholinergic signaling has been shown to affect CFTR function but this is the first time CFTR loss of function has been shown to alter cholinergic signaling. Functional assays confirm that cholinergic sensitivity is diminished in CF guts and restoring cholinergic signaling via *Ace* knockdown rescues multiple CF-associated phenotypes. Furthermore, we identify the transcription factor Forkhead (Fkh), the *Drosophila* homolog of human FOXA1/FOXA2, which is known to be a positive regulator of *Cftr* in the intestine, as a positive regulator of *Ace* expression in CF guts. This study establishes the *Drosophila* gut as a powerful model to investigate CF pathogenesis, genetic modifiers, and identifies Ace and Fkh as genetic modifiers. This work also suggests that enhancing cholinergic signaling may represent a viable therapeutic strategy for gastrointestinal manifestations of CF.

## Introduction

Cystic Fibrosis (CF) is a genetic disease that affects approximately 1 in 2,500 newborns in the United States^1^. CF is caused by mutations in the Cystic Fibrosis Transmembrane conductance Regulator (CFTR) chloride channel, which is expressed in certain epithelia, including the respiratory tract, pancreas, and the gut^2-4^. The CF phenotype is most closely associated with the accumulation of mucus in the pulmonary and gastrointestinal tracts, which can lead to inflammation, bacterial infection, and malnutrition^1,5-8^. Much of CF research has focused on the lung, as the primary cause of death in CF patients are related to lung complications, however, CF causes pathologies in other CFTR-expressing tissues, including the gut, that result in various clinical phenotypes^7,9-13^. For example, many CF patients are born with intestinal blockage (Meconcium Ileus) and later in life have decreased intestinal motility, dysbiosis, inflammation, and poor nutrient absorption in the gut^7,9-13^. Additionally, as life expectancy has increased following better treatments, complications in the gastrointestinal tract have become an increasing cause of morbidity^10-15^. These complications include small bowel bacterial overgrowth, bowel obstructions, and increased risk of intestinal cancers^11-13^. Recent studies have suggested that CF modulator drugs may not fully correct the inflammation and dysbiosis seen in the gut of CF patients and that CF patients on modulator drugs still report GI symptoms^14,16-19^. Therefore, additional therapies targeting the gut, not directly related to modulating CF activity may be useful for quality of life for patients with CF. Recently, there have been studies highlighting how the CF gut can crosstalk with many other organ systems, further highlighting the importance of investigating how loss of CFTR function effects gut biology^20-22^.

The *Drosophila* ortholog of human *Cftr* has recently been identified and used to establish an intestinal CF model^23^. This model revealed that *Cftr* knockdown in the *Drosophila* intestine disrupts osmotic homeostasis and displays CF-like phenotypes in the intestinal epithelium^23^. While this work introduced *Drosophila* as a CF model, the phenotypes examined were largely cellular phenotypes and not clinical manifestations of CF. Here, we further characterize the CF model gut, demonstrating that several clinical pathologies, including reduced intestinal motility and nutrient malabsorption are preserved. Furthermore, the *Drosophila* CF model displays characteristics consistent with a failure to thrive phenotype. Additionally, we perform single nuclei RNA-seq (snRNA-seq) to further characterize our CF gut model and learn new biology relevant to CF. Interestingly, we found increased *acetylcholine esterase (Ace)* expression in CF model guts, which results in reduced cholinergic signaling potential. Recent work in *Drosophila* has demonstrated that cholinergic signaling is important for maintenance of the intestinal epithelia barrier and is required for the intestinal epithelium to return to homeostasis after injury^24,25^. When Ace is upregulated after injury in enterocytes the intestine is unable to make a return to homeostatic conditions^24^. Additionally cholinergic signaling has been implicated in other diseases of intestinal inflammation such as IBD but has not yet been investigated in the context of the CF intestine^26,27^. Previous studies have demonstrated that cholinergic signaling can increase CFTR function^28-31^ but this is the first work that indicating that CFTR function can have a reciprocal effect on cholinergic signaling. We further show the decrease in cholinergic signaling observed in the CF model guts may be clinically relevant as restoring sensitivity to cholinergic signaling rescues many CF pathologies in *Drosophila.* Finally, we identify Forkhead (Fkh), a FOXA1/A2 homolog as a transcriptional regulator of *Ace* expression in the CF model gut. FOXA1/A2 is a pioneering transcription factor that positively regulates the expression of *Cftr* and other transmembrane proteins and ion channels important for regulating ion homeostasis in the intestinal epithelia^32,33^. Altogether, our findings help establish *Drosophila* as a model organism to study GI manifestations of CF. We identify Ace (AChE) and Fkh (FOXA1/A2) as genetic modifiers of CF and our findings suggests that the cholinergic signaling pathway may be a viable therapeutic target in CF gastrointestinal disease.

## Results

### Knockdown of *Cftr* in the *Drosophila* midgut recapitulates clinical pathologies of Cystic Fibrosis

**Figure 1.**
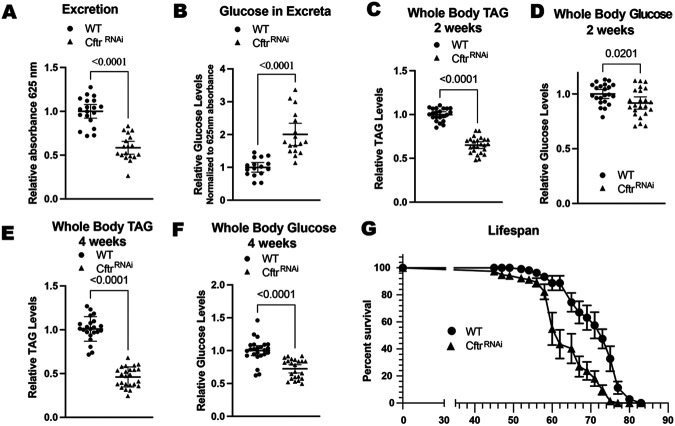
CF model gut recapitulates hallmarks of CF **(A)** CF model guts have decreased excretion rate compared to WT guts. n= 19 vials of 10-15 females from 3 independent experiments. **(B)** CF model guts have increased glucose in excreta compared to WT flies. n= 16 (WT), 17 *(Cftr^RNAi^* vials of 15-20 females from 3 independent experiments. **(C-F)** CF model guts have reduced whole body energy stores to WT flies. **(C)** CF model guts have reduced TAG levels at 2 weeks of age compared to WT flies. n= 22 (WT), 24 (*Cftr*^*RNAi*^) of 5 pooled females from 4 independent experiments. **(D)** CF model guts have slightly reduced whole body glucose levels at 2 weeks of age compared to WT flies. n= 22 (WT), 24 (*Cftr*^*RNAi*^) of 5 pooled females from 4 independent experiments. **(E)** CF model gut flies have reduced whole body TAG levels at 4 weeks of age compared to WT flies. n= 22 (WT), 24 (*Cftr^RNAi^*) of 5 pooled females from 4 independent crosses. **(F)** CF model gut flies have reduced whole body glucose at 4 weeks of age compared to WT flies. n= 24 (WT), 23 (*Cftr*^*RNAi*^) of 5 pooled females from 4 independent crosses. **(A-F)** p values were calculated using the Mann-Whitney test in GraphPad prism. Error bars are mean with 95% CI. **(G)** CF model gut flies have reduced lifespans compared to WT flies. n= 9 (WT), 12 (*Cftr*^*RNAi*^) vials with 10-15 females from 2 independent crosses, error bars are mean +/− SEM. **(A-G)** WT is *Myo1A > +* and *Cftr*^RNAi^ is *Myo1A >UAS-Cftr*^*RNAi*^.

Cystic Fibrosis (CF) patients display a range of multiple organ specific as well as systemic pathologies, including gastrointestinal complications^10-13,34-36^. Therefore, we examined whether CF model guts, with enterocyte-specific knockdown of *Cftr (Myo1A-Gal4 > UAS-Cftr*^*RNAi*^) recapitulated typical clinical pathologies. CF patients are at increased risk for gastrointestinal cancers and microbial dysbiosis ^11,13,36,37^. These clinical observations are consistent with previous findings in the *Drosophila* CF model, showing gut hyperplasia and increased bacterial load^23^. Additional common CF gut pathologies such as meconium ileus, distal intestinal obstruction syndrome, and constipation are linked to impaired intestinal motility ^9,35,38^. To test whether the CF model exhibits reduced gut motility, we performed an excretion assay. *Cftr* knockdown flies showed significantly reduced excretion compared to wild-type (WT) controls, indicating impaired intestinal transit (Fig. 1A; S1A). Malabsorption of nutrients in the intestine is another hallmark of CF^39^. In line with this, CF model flies exhibited increased glucose levels in excreta, suggesting reduced glucose absorption in the intestine (Fig. 1B; S1B).

Failure to thrive, where patients with CF fail to gain weight and grow at the expected rate, is a more systemic pathology in CF patients with a strong intestinal component^7,39,40^. *Cftr* knockdown flies have significantly reduced energy stores, with lower levels of whole body triacyl glycerides (TAGs) and glucose at both 2 and 4 weeks of age (Fig 1 C-F, S1 C-F). The reduction of TAG levels is robust at both 2 and 4 weeks of age while the reduction of glucose stores is more apparent at 4 weeks of age. This reduction in whole body energy stores is not due to a developmental defect as inducing *Cftr* knockdown in adult flies using the temperature sensitive Gal80^ts^ repressor also leads to reduced whole body TAGs and glucose (S1 G-H). This reduction in whole body energy stores is consistent with a failure to thrive phenotype. As the CF gut model only has reduced *Cftr* expression in the midgut, it may be useful in studying gut-specific contributions to the failure-to-thrive phenotype.

Finally, CF patients have reduced lifespans, although they have improved dramatically in recent years due to the availability of better treatments^41^. Our model fly shows that loss of *Cftr* in the gut alone is sufficient to reduce the lifespan of the fly, highlighting the importance of CFTR function in the gut (Fig 1G, S1I). Overall, these results show that loss of *Cftr* in the enterocytes recapitulates many hallmarks of CF in the gastrointestinal system.

### Single nuclei analysis of CF model guts

**Figure 2.**
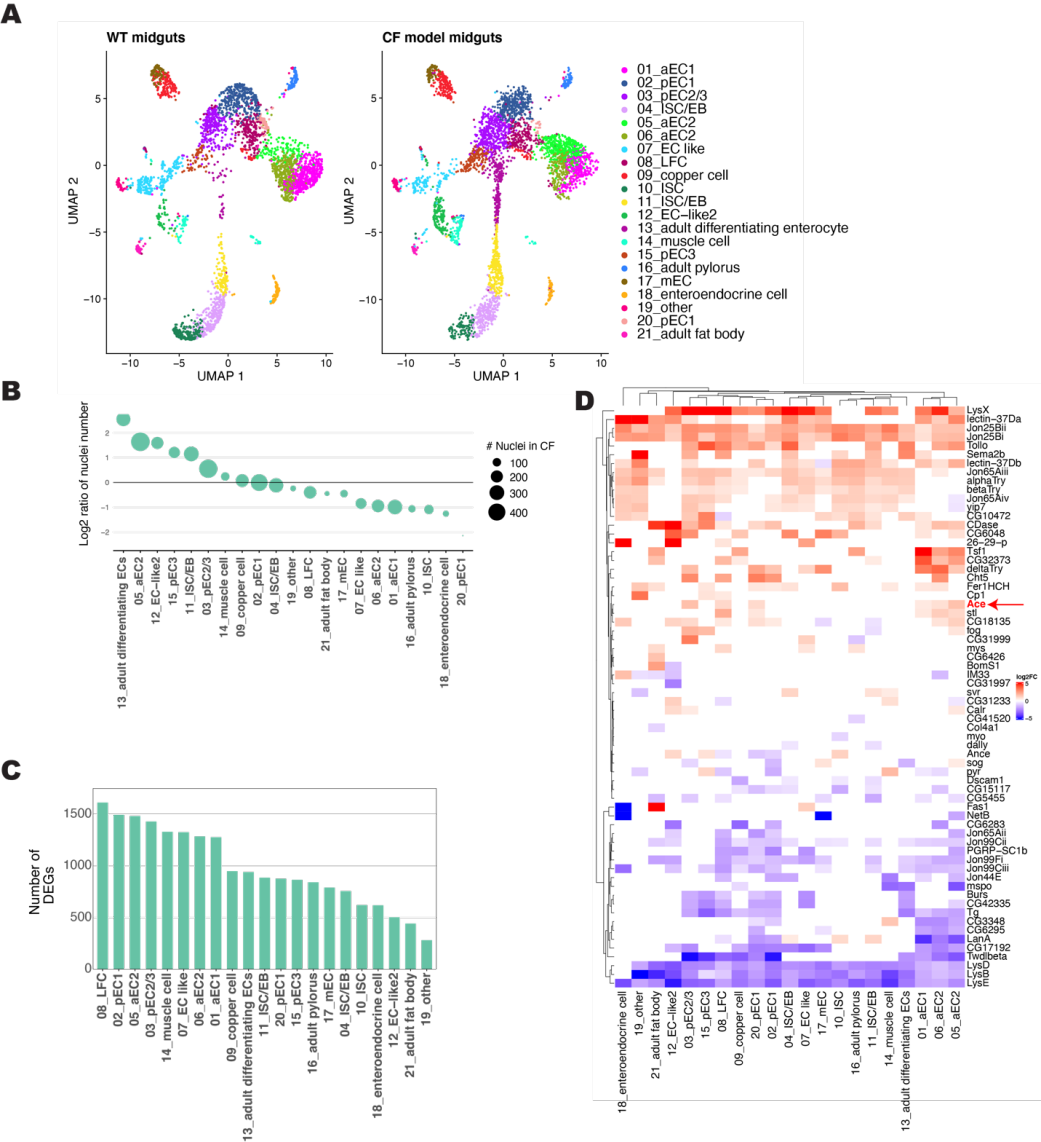
snRNA-Seq of CF model guts **(A)** Annotated cell clusters of snRNA-Seq from WT *(Myo1A> +)* and CF *(Myo1A>Cftr*^*RNAi*^) midguts, visualized with UMAP. **(B)** Over and underrepresented cell clusters in CF model gut snRNA-seq data set. Size of nuclei represent number of nuclei in CF model gut analysis for indicated cluster. **(C)** Number of differentially expressed genes in each cell cluster between WT and CF model gut snRNA-seq data sets. **(D)** Heat map of the top 5 differentially expressed secreted proteins for each cell type. Color reflects the log2fold change in expression in CF model guts comparing to control per cell cluster.

To further characterize the CF gut model, we performed snRNA-seq on the midgut of WT and CF model gut flies. The single nuclei analysis recovered 3,669 nuclei for WT and 4,166 for CF midguts that were clustered into 21 clusters annotated using the marker genes from the previously published cell atlas of the *Drosophila* gut^42,43^ (Fig 2A). UMAP analysis does not identify any unique clusters in the CF model guts, however there are a number of clusters that are over or under-represented in the CF model (Fig 2A-B, S2A). This includes increased intestinal stem cells (ISCs), which is consistent with the hyperplasia previously described in the CF model gut^23^. Furthermore, there are a significant number of differentially expressed genes (DEGs) between WT and CFTR deficient guts across all identified cell clusters (Fig 2C, Supplementary File S1). Together these results indicate substantial differences in the transcriptional profile and cellular composition between WT and CF model guts.

Interestingly, many secreted peptides are differentially expressed indicating that the CF gut model may be a good model to study the crosstalk between the CF gut and other organs (Fig 2D, S2B, Supplementary File S2). Among the upregulated secreted proteins in the CF model midguts are several that have also been identified as key factors secreted by Yki-activated gut tumors involved in tumorigenesis and communication with other organs (Impl2, Pvf1, Itp, and Upd3)^44,45^ (S2C). This overlap suggests that the CF model gut may exhibit a predisposition toward intestinal tumorigenesis similar to human patients with CF who are at increased risk of gastrointestinal cancers^11,13,36,37^. Additionally, among the secreted peptides, there are differences in the expression of *Drosophila* mucin genes. Consistent with known CF pathophysiology of increased mucin production^23,46^ there is overall an increase in the expression of mucin genes (S2D).

**Figure 3.**
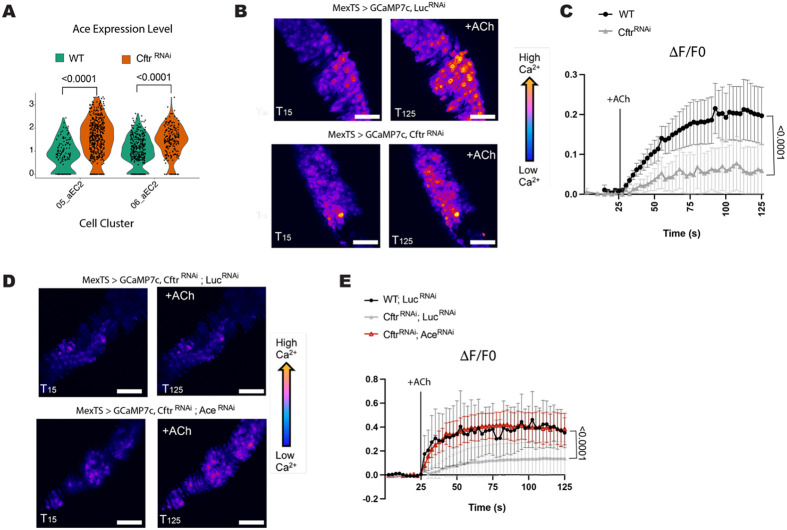
CF model guts have reduced sensitivity to cholinergic signaling **(A)** Violin plot of acetylcholine esterase *(Ace)* expression in anterior EC2 cell clusters indicates higher *Ace* expression in CF model guts. Each dot represents expression in a single-nucleus, n=137 (WT) or 479 *(Cftr^RNAi^*) for 05_aEC2 and n=362 (WT) or 213 *(Cftr*^*RNAi*^) for 06_aEC2. pValues were calculated using the Mann-Whitney test in ggplot2. **(B-C)** *Cftr* deficient midguts have reduced sensitivity to acetylcholine (ACh) stimulation as measured by calcium levels in enterocytes. **(B)** Representative images of GCaMP7c fluorescence, in WT *(MexTS > GCaMP7c, LucRNAi)* and *Cftr* deficient guts *(MexTS > GCaMP7c, Cftr^RNAi^*) before (T15s) or after addition of ACh (T125). Scale bar 50 μ.m. **(C)** Graph of average relative fluorescent intensity, ΔF/F0, per frame (2.5s per frame) and genotype. n= 9 (WT) and 10 *(Cftr*^*RNAi*^) from 4 independent experiments. Error bars are mean +/− SEM. pValue was calculated using the Mann-Whitney test in GraphPad prism. **(D-E)** Ace knockdown increases sensitivity to ACh stimulation in *Cftr* deficient enterocytes. **(D)** Representative images of GCaMP7c fluorescence, in *Cftr* deficient *(MexTS > GCaMP7c, Cftr*^*RNAi*^; Luc^*RNAi*^) midguts and *Cftr* deficient guts with *Ace* knockdown (*MexTS > GCaMP7c, Cftr*^*RNAi*^*; Ace*^*RNAi*^) before (T15s) or after addition of ACh (T125). Scale bar 50 μ.m. **(E)** Graph of average relative fluorescent intensity, ΔF/F0, per frame (2.5s per frame) and genotype. n= 3 (WT), 5 *(Cftr*^*RNAi*^, *Luc*^*RNAi*^), 4 *(Cftr*^*RNAi*^, *Ace*^*RNAi*^) from 3 independent experiment. Error bars are mean +/− SEM. pValues were calculated using 2-way ANOVA with Tukey’s multiple comparisons main column effect in GraphPad prism.

In addition to the expected increase of cells in the ISCs cell clusters there is a redistribution of cells in anterior and posterior EC cell clusters (Fig 2A-B, S2A). In the CF preferred anterior EC cluster, one of the top 5 differentially expressed secreted peptides is *acetylcholine esterase (Ace)* (Fig 2D). *Ace* expression is increased overall in CF model guts but is particularly apparent in the anterior EC clusters (Fig 3A, S3A-B). We confirmed increased *Ace* expression in our CF model guts by qPCR analysis of whole guts (S3C).

Ace hydrolyzes acetylcholine into acetate and choline thereby inhibiting cholinergic signaling. Cholinergic signaling has recently been shown to be required for recovery of the intestinal epithelium after damage and for maintaining intestinal barrier function in Drosophila^24,25^. Furthermore, cholinergic signaling has been shown to increase CFTR activity but there are no studies showing CFTR function affecting cholinergic signaling^28-31^. Therefore, we investigated the importance of increased *Ace* expression in our CF gut model.

### Reduced Cholinergic signaling in the CF Gut Model

Cholinergic signaling modulates ion transport in the mammalian intestinal epithelium in a Ca^2+^-dependent manner^47^. As Ace degrades acetylcholine (ACh), the agonist of cholinergic receptors, increased *Ace* expression decreases sensitivity to cholinergic signaling. To test for cholinergic sensitivity in the *Cftr* deficient midguts we visualized Ca^2+^ by conditionally expressing the Ca^2+^ indicator GCaMP7c^48^ in ECs and performed *ex vivo* live imaging^24^. We found that Ca^2+^ levels in CF model guts were reduced in response to ACh, indicating that CF model guts had a dampened response to ACh stimulation compared to WT guts (Fig 3B-C).

Cholinergic receptors include muscarinic and nicotinic receptors which can be activated by muscarine chloride and nicotine, respectively. Stimulation of the gut with muscarine chloride had very low response even in the WT indicating that muscarinic receptors may not be active in the *Drosophila* gut under homeostatic or CF-like conditions (Data not shown). Nicotine treatment which activates nicotinic receptors without being degraded by Ace increases Ca^2+^ levels in WT and to a lesser extent in CF model guts (S3D-E). To test the importance of *Ace* expression in the CF gut models sensitivity to cholinergic signaling we used the Mex-Gal4 (enterocyte specific driver) together with the Gal4 repressor Tubulin-Gal80^TS^ to drive expression of both *Ace*^*RNAi*^ and *Cftr*^*RNAi*^ in adult *Drosophila* for 2 weeks (S4). When *Ace* levels are reduced in the CF background the response to ACh is increased to WT levels (Fig 3D-E). This result indicates that reducing *Ace* expression in CF model guts is sufficient for rescuing sensitivity to cholinergic signaling. Therefore, if decreased cholinergic signaling is important for CF pathophysiology reducing *Ace* expression should alter CF phenotypes in the *Drosophila* model.

### Increasing cholinergic signaling in CF model gut rescues several CF phenotypes

**Figure 4.**
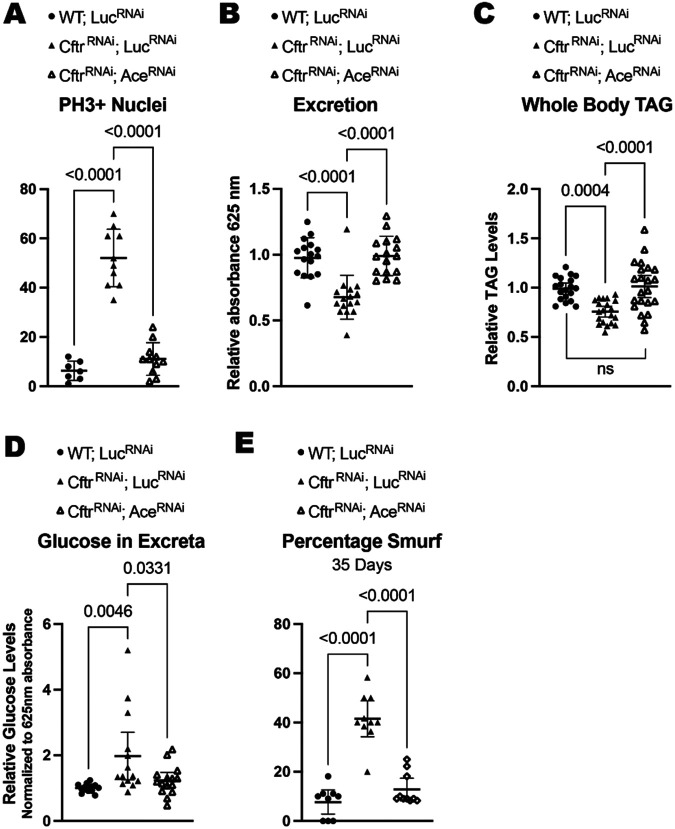
Increasing cholinergic signaling rescues CF phenotypes **(A)** *Ace* knockdown rescues the increased proliferation seen in CF model guts. n=7 (WT; *Luc*^*RNAi*^), 10 (*Cftr^RNAi^*), 11 (*Cftr*^*RNAi*^*; Ace*^*RNAi*^). **(B)** Ace knockdown increases intestinal motility as measured by excretion rate in CF model guts. n=16 vials with 9-13 female flies from 3 independent experiments. **(C)** *Ace* knockdown rescues whole body TAG levels at 2 weeks of age in CF model guts. n=19 (WT; *Luc*^*RNAi*^), 20 *(Cftr*^*RNAi*^*, Luc*^*RNAi*^), 22 *(Cftr*^*RNAi*^*; Ace*^*RNAi*^) of 5 pooled females from 4 independent experiments. **(D)** *Ace* knockdown reduces the amount of glucose remaining in excreta in CF model gut flies. n=14 (WT; *Luc^RNAi^*), 14 *(Cftr*^RNAi^, *Luc^RNAi^* 15 *(Cftr^RNAi^*; *Ace*^*RNAi*^) vials of 15-20 females from 3 independent experiments. **(E)** CF model gut flies have increased intestinal permeability, measured by the presence of normally non-permeable blue dye in the hemolymph at 35 days of age, which is rescued by *Ace* knockdown. n=9 (WT; *Luc*^*RNAi*^), 10 *(Cftr*^*RNAi*^*, Luc*^*RNAi*^), 10 *(Cftr*^*RNAi*^; *Ace*^*RNAi*^) vials of 9-13 females from 3 independent experiments. **(A-E)** p values were calculated using ordinary one-way ANOVA with Tukey’s multiple comparisons test in GraphPad prism. Error bars are mean with 95% CI. **(A-E)** RNAi constructs were expressed in enterocytes using the enterocyte specific driver *Mex*^*TS*^ *(Mex-Gal4* with the temperature sensitive gal4 repressor *tubulin gal80*^*TS*^) (S4).

Recent work has demonstrated that cholinergic signaling is required for the gut to return to homeostasis after damage and that loss of cholinergic signaling after damage leads to increased proliferation of ISCs^24^. Therefore, we examined whether *Ace* expression affected the hyperplasia present in the CF model gut. Indeed, *Ace* knockdown in the CF model gut reduced proliferation in the gut indicating that decreased cholinergic signaling contributes to the increased proliferation observed in the CF intestine (Fig 4A).

We also found that increasing cholinergic signaling by reducing *Ace* levels in the CF model gut rescued the excretion rate indicating that cholinergic signaling can regulate intestinal transit in the CF model (Fig 4B). Reducing *Ace* expression in the CF model gut was also sufficient for rescuing both the whole-body TAG stores and the malabsorption phenotypes observed in the CF model gut at 2 weeks of age (Fig 4C-D).

Recently cholinergic signaling has been linked to intestinal barrier function in the *Drosophila* gut^25^. As there is evidence of increased intestinal permeability in patients with CF^49^ we tested whether the CF model gut had decreased intestinal barrier function. To test intestinal barrier function, we performed a Smurf assay, where flies are fed a blue food dye that in healthy guts is impermeable to the intestinal barrier. When intestinal barrier function is impaired the blue dye leaks into the hemolymph and the fly turns blue (Smurf)^50^. We found that CF model guts have decreased barrier function demonstrated by the increase percentage of Smurf flies at 35 days of age (Fig 4E). Importantly, intestinal barrier integrity is rescued by increasing cholinergic signally via *Ace* knockdown in the gut (Fig 4E).

Altogether these results indicate that the reduction in cholinergic signaling observed in CF model guts contributes to multiple CF pathologies.

### Fkh regulates *Ace* transcription in CF model gut

**Figure 5.**
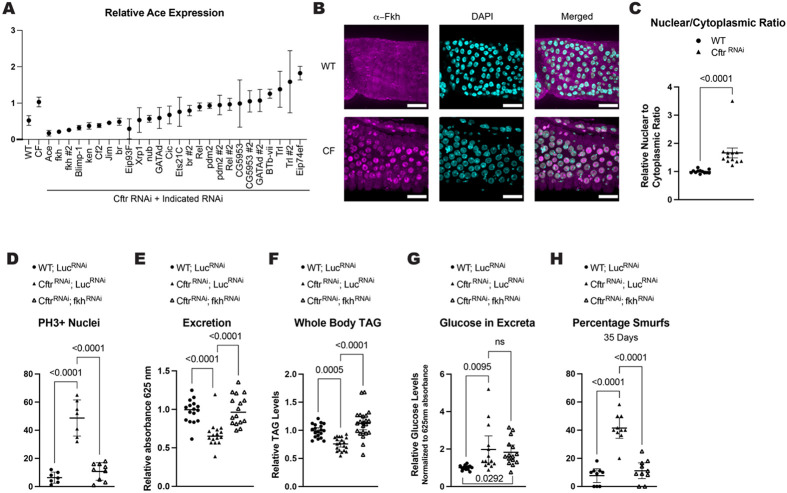
Fkh regulates *Ace* transcription and CF pathologies in the CF model gut **(A)** Screen of transcription factors identifies Fkh as a candidate transcription factor of *Ace* in the CF model guts. n=2 biological replicates of 15 pooled guts. error bars are mean +/− SD. RNAi constructs were expressed in adult flies for 2 weeks using *Myo*^*TS*^ *(Myo1A-Gal4, tub-Gal80*^*TS*^) driver (S5A). **(B)** Representative images of increased Fkh nuclear localization in CF model guts compared to WT. scale bars 25μm. **(C)** Fkh has increased nuclear localization in *Cftr* deficient guts. n=12 from 4 independent experiments. p values were calculated using the Mann-Whitney test in GraphPad prism. Error bars are mean with 95% CI. **(D)** *fkh* knockdown rescues the hyperplasia observed in CF model guts. n= 7 (WT; *Luc*^*RNAi*^), 9 *(Cftr^RNAi^*, *Luc*^*RNAi*^), 9 *(Cftr*^*RNAi*^*; fkh*^*RNAi*^) from 2 independent experiments. **(E)** *fkh* knockdown increases intestinal motility as measured by excretion rate in CF model guts. n=16 vials with 9-13 female flies from 3 independent experiments. **(F)** *fkh* knockdown rescues whole body TAG levels at 2 weeks of age in CF model guts. n=19 (WT; Luc^RNAi^), 20 *(Cftr*^*RNAi*^*, Luc*^*RNAi*^), 22 *(Cftr*^*RNAi*^*; fkhR*^*RNAi*^) of 5 pooled females from 4 independent experiments. **(G)** *fkh* knockdown does not rescue the malabsorption of *Cftr* deficient guts. n=14 (WT; *Luc*^*RNAi*^), 14 *(Cftr*^*RNAi*^*, Luc*^*RNAi*^), 15 *(Cftr*^*RNAi*^*; fkhR*^*RNAi*^) vials of 15-20 females from 3 independent experiments. **(H)** Fkh depletion rescues intestinal barrier function in flies with *Cftr* deficient enterocytes as assessed by percentage smurf flies at 35 days of age. n=9 (WT; *Luc*^*RNAi*^), 10 *(Cftr*^*RNAi*^, *Luc*^*RNAi*^), 10 *(Cftr*^*RNAi*^; *fkhR*^*RNAi*^) vials of 9-13 females from 3 independent experiments. **(D-H)** pValues were calculated using ordinary one-way ANOVA with Tukey’s multiple comparisons test in GraphPad prism. Error bars are mean with 95% CI. **(D-H)** Share values for WT; *Luc*^*RNAi*^ and *Cftr*^*RNAi*^; *fkhR*^*RNAi*^ with Figure 4 A-E as Ace and *fkh* knockdown in CF background were performed at the same time and used the same controls. **(D-H)** RNAi constructs were expressed in enterocytes using the enterocyte specific driver *Mex*^*TS*^ *(Mex-Gal4* with the temperature sensitive gal4 repressor *tubulin gal80^TS^*)(S4).

As *Ace* RNA levels are changed in the CF model gut, we performed a screen of transcription factors to identify how *Ace* expression is regulated. To be included in the screen the transcription factor had to 1) have expression in the single cell data where *Ace* is expressed (S5A) and 2) have potential binding sites as assessed by the TF2G database (Table S1)^51^. We identified 18 putative transcription factors (TFs) and used RNAi to test their ability to regulate *Ace* transcription in the *Cftr* deficient background *(MyoTS> Cftr^RNAi^, TF^RNAi^*) by quantifying *Ace* levels (S5B). The top 2 hits with the lowest *Ace* expression in the CF background were *forkhead (fkh)* RNAi lines (Fig 5A). In *Drosophila* Fkh has been shown to regulate ISC proliferation and expression of nutrient transporters in the intestine^52,53^. Interestingly, the mammalian ortholog FOXA1/A2 regulate expression of CFTR and other transmembrane proteins important for regulating ion homeostasis in the intestinal epithelium^32,33^. Furthermore, Fkh is one of only 3 TFs that has potential binding sites identified by both motif analysis and Chip-seq data near the *Ace* gene (Table S1). Additionally, *fkh* expression has the highest correlation with *Ace* expression in the aEC2 CF cell cluster of the transcription factors we tested (S5C). Therefore, we investigated Fkh activity and function in the CF model guts.

As there is minimal difference in the expression of *fkh* between WT and CF midguts in our single cell data and that Fkh activity is known to be regulated by nuclear translocation^53,54^, we measured Fkh nuclear localization. We used confocal microscopy to image the localization of Fkh in the anterior midguts, the region with highest *Ace* expression in the *Cftr* deficient guts. We found increased nuclear localization of the Fkh protein in the nuclei of CF model compared to WT guts consistent with increased Fkh transcriptional activity (Fig 5B-C).

If Fkh is regulating *Ace* transcription, we expect Fkh manipulation to also modulate CF pathologies. Indeed, reducing *fkh* expression lowered ISC proliferation, increased intestinal motility, increased whole body TAG stores, and increased intestinal barrier integrity in the CF model guts (Fig 5D,E,H). Interestingly, *fkh* knockdown in the *Cftr* deficient background does not rescue the nutrient malabsorption phenotype as shown by no change in the amount of glucose remaining in the excreta (Fig 5F). This is the one phenotype tested that does not phenocopy *Ace* knockdown, likely due to other transcriptional targets of Fkh^45^. These results indicate that increased Fkh nuclear localization downstream of CFTR loss of function regulates CF pathophysiology likely through increasing *Ace* expression.

## Discussion

We show that the *Drosophila* CF intestinal model recapitulates several clinical pathologies of CF, including poor intestinal motility, nutrient malabsorption, reduced whole body energy stores, and decreased intestinal barrier function. Additionally, we performed snRNA-seq on *Cftr* deficient and WT midguts and uncovered in *Cftr* deficient enterocytes an upregulation of *acetylcholine esterase (Ace)* expression. This upregulation in *Ace* results in a decreased sensitivity to acetylcholine in *Cftr* deficient enterocytes. Importantly, we demonstrate that increasing cholinergic sensitivity in *Cftr* deficient enterocytes by *Ace* knockdown rescues several clinical pathologies of CF. Finally, we identify the FOXA1/A2 ortholog Fkh as a putative transcription factor for *Ace,* that has increased nuclear localization in the *Cftr* deficient midguts.

Previous work has shown that cholinergic signaling can increase CFTR activity^28,29^ , but it has not been shown previously that CFTR can affect cholinergic signaling. Here, we demonstrate that loss of CFTR function leads to an increase in *Ace* expression and a reduction in the guts sensitivity to cholinergic signaling indicating that there may be reciprocal regulation between cholinergic signaling and CFTR activity. This is interesting as it has been recently demonstrated in *Drosophila* that cholinergic signaling is required for a return to homeostasis after damage to the gut^24^. The increased *Ace* expression and lack of cholinergic signaling potential may be prevent the gut to return to homeostasis from the damage being done to the gut epithelium by loss of CFTR. This may be because the CF gut is constantly being damaged and it may be energetically unfavorable to be constantly working to return to homeostasis, but further studies are required to explore this hypothesis. Additionally, we demonstrate that increasing the sensitivity to cholinergic signaling in CF model guts rescues many pathological phenotypes. Cholinergic signaling is already being investigated as a therapeutic target for diseases of intestinal inflammation such as IBD^26,27,55^ which shares many GI symptoms with CF ^56,57^ This work suggests AChE inhibition may also be therapeutically beneficial for patients with CF as well.

We also show that *Ace* transcription downstream of *Cftr* loss of function is at least in part regulated by an increase in Fkh activity. Interestingly, the human orthologs of Fkh FOXA1/FOXA2 are known to regulate the expression of CFTR as well as other ion channels important for intestinal cell function^32,33^. Therefore, in the context of ECs with decreased *Cftr* activity increasing FOXA1/FOXA2 activity could be a compensatory mechanism to try and increase CFTR function. While knockdown of *fkh* largely phenocopied *Ace* knockdown, loss of *fkh* expression did not rescue the poor nutrient absorption phenotype. This may be because Fkh also regulates the transcription of many nutrient transporters ^52^. Intriguingly, even though nutrient absorption is not rescued whole body TAG levels are increased. This decouples the nutrient absorption phenotype from the whole-body energy stores which could be an interesting model to study. Another interesting follow up study not addressed in this study would be to examine how changes in CFTR function results in an increase in Fkh nuclear translocation and transcriptional activity. Finally, it is important to test if this interaction between CFTR loss of function, increased Fkh activity and increased *Ace* expression is conserved in mammalian systems as Fkh and Ace could be therapeutically relevant targets.

We performed single-nucleus RNA sequencing (snRNA-Seq) to compare the intestines of wild-type (WT) and the cystic fibrosis (CF) *Drosophila* model. While no unique cell clusters were identified in the CF model, we observed an increase in intestinal stem cells (ISCs) and newly differentiated enterocytes (ECs), along with a shift in EC cluster composition. The expansion of the stem cell population aligns with established CF biology, and we also detected changes in genes such as mucins, consistent with phenotypes seen in human CF. We further investigated the upregulation of *Ace* in the CF gut, prompted by recent studies highlighting the role of cholinergic signaling in maintaining intestinal epithelial homeostasis in *Drosophila.* In addition, we identified a substantial number of differentially expressed genes (DEGs) across all cell types, presenting numerous opportunities for future investigation. Notably, many differentially expressed secreted peptides may play a role in inter-organ communication. Together with observed changes in whole-body metabolite levels and lifespan, these findings suggest that the Drosophila CF model may provide valuable insights into how gut dysfunction influences systemic physiology.

Our study further establishes *Drosophila* as a useful model to study CF. Importantly, since CF clinical outcomes are only partially determined by CFTR mutations and are influenced by other genetic modifiers, as shown in previous studies^58-61^, this highlights the potential of *Drosophila* for investigating CF genetic modifiers—especially those that are challenging to study at scale in other CF models. Many of these modifiers have been identified by GWAS studies while others are hypothesized based on interaction with CFTR or other experimental evidence^58-61^. However, in most cases, these putative modifiers have not been validated and studied in detail^58-61^. The fly model can be used to screen which of these potential genetic modifiers alter CF pathophysiology. Understanding how CF modifiers affect physiology could lead to new drug targets that could be beneficial to all CF patients regardless of the type of CFTR mutations. Similarly, *Drosophila* would be an ideal model for a first pass to screen chemical libraries for potential CF therapeutics as a relatively cheap *in vivo* model with clinically relevant phenotypes.

## Methods

### Fly lines

The following fly stocks were used in this study: Drivers from Perrimon lab stocks: mex1-Gal4^62^ (mex), mex1-Gal4 Tubulin-Gal80TS (*mex*TS), Myo31DF^NP0001^-Gal4 (myo1A-Gal4)^63^, Myo1A-Gal4 Tubulin-Gal80TS(*myo1A*TS).

UAS-RNAi lines from NIG and BDSC Drosophila Stock Centers: UAS-Cftr^RNAi^- NIG Stocks: 5789R-1 & 5789R-4, UAS-Ace^RNAi^- BDSC 25958, UAS-Luciferase^RNAi^-BDSC 31603 UAS-fkh^RNAi^- BDSC 27072, UAS-fkh^RNAi^ #2- BDSC 58059, UAS-Blimp-1^RNAi^- BDSC57479, UAS-Ken^RNAi^- BDSC-34739, UAS-CF2^RNAi^- BDSC 57256, UAS-Jim^RNAi^- BDSC 42662, UAS-br^RNAi^- BDSC 33641, UAS-Eip93F^RNAi^ BDSC 57868, UAS-Xrp1^RNAi^- BDSC 51054, UAS-nub^RNAi^- BDSC 28338, UAS-GATAd^RNAi^- BDSC 34640, UAS-Cic^RNAi^- BDSC 25995, UAS-Ets21c^RNAi^- BDSC 39069, UAS-br^RNAi^ #2- BDSC 27172, UAS-rel^RNAi^- BDSC 28943, UAS-pdm2^RNAi^- BDSC 29453, UAS-pdm2^RNAi^ #2- BDSC 50665, UAS-rel^RNAi^ #2- BDSC 35661, UAS-CG5953^RNAi^- BDSC 57543, UAS-CG5953RNAi #2- BDSC 57287, UAS-GATAd^RNAi^-BDSC 33747, UAS- BTb-viiRNAi- BDSC 28912, UAS-Trl^RNAi^- BDSC 40940, UAS-TrlRNAi #2- BDSC 41582, UAS- Eip74ef- BDSC 29353.

### Excretion assay

Flies were fed overnight on lab food with 2 g/100 ml FD&C Blue Dye (12-15 flies per vial for female and 15-20 flies per vial for male). After overnight feed they were moved to 5 ml culture tube without food for 2 hrs (female) or 1 hr 45 minutes (males). Excreta was collected in 400 ml of .05% PBST and 100 ml was used in triplicate to measure blue absorbance at 625 nm using the SpectraMax Paradigm Multi-mode microplate reader (Molecular Devices). A standard curve was made with serial dilutions of the FD&C Blue Dye in .05% PBS and values were normalized to WT for each experiment.

### Whole body metabolite measurements

For each replicate 5 flies (females) or 8 flies (males) were collected in eppendorph tubes containing 1.0 mm Zirconium Oxide Beads (Next Advance Lab Products—ZROB10) and frozen on dry ice and stored at −80C. Samples were moved to ice and 500 μL of ice-cold PBS with 0.05% Triton-X-100(Sigma) was added to the tubes. Homogenization was carried out using a TissueLyser II (QIAGEN) for 2-3 cycles of 30 seconds each, at an oscillation frequency of 30 Hz/s. Tubes were centrifuged for 1 minute at 3500 g to remove debris, and the homogenate was immediately used for glucose, protein, and TAG quantification. Quantifications were performed using a SpectraMax Paradigm Multi-mode microplate reader (Molecular Devices). For protein measurements, 5 μL of the homogenate was mixed with 200 μL of the BCA Protein Assay Kit in triplicate (Pierce BCA Protein Assay Kit), followed by incubation for 30 minutes at 37°C with gentle shaking in 96-well Microplates (Greiner Bio-One) and measuring absorbance at 562nm. Glucose quantification involved mixing 10 μL of homogenate with 100 μL of Infinity Glucose Hexokinase Reagent in triplicate (Thermo Fisher Scientific—TR15421), incubating for 30 minutes at 37°C in 96-well Microplates UV-Star (Greiner Bio-One – 655801) with gentle shaking and measuring 340nm. For triglycerides, 5 μL of the homogenate was mixed with 150 μL of Triglycerides Reagent in triplicate (Thermo Fisher Scientific—TR22421) and incubated for 10 minutes at 37°C in 96-well Microplates (Greiner Bio-One) with gentle shaking and absorbance was measured at 520 nm. Values were calculated from standard curves using seven serial dilutions (1:1) of BSA (Pierce BCA Protein Assay Kit), glycerol standard solution (Sigma), or glucose standard solution (Sigma). Glucose and TAG levels were normalized to BCA protein levels and then normalized to WT levels for each independent cross.

### Glucose in excreta

Flies were fed overnight on lab food supplemented with 1 g/ 100 ml FD&C Blue dye with 15-20 flies per vial. Flies were transferred to 5 ml culture tubes with 200 ul of lab food supplemented with 1 g/ 100 ml FD&C Blue Dye for 6 hr. Excreta was collected in 200 ul of .05% PBST and transferred to a 96 well microplate (Greiner Bio-One – 655801) and absorbance was measured at 625 nm using the SpectraMax Paradigm Multi-mode microplate reader (Molecular Devices). A standard curve was made with serial dilutions of the FD&C Blue Dye in .05% PBS. Glucose quantification was performed as in whole body metabolite measurements and glucose levels were normalized to total excreta amount (625 nm) absorbance before being normalized to the WT condition in each experiment.

### Lifespan assay

15 (females) or 20 (males) 2-4 day old adult flies were placed in vials on standard laboratory food. Flies were flipped every other day and vials were scored for percent survival.

### Smurf assay

Smurf assay was performed as previously described^50^. Briefly 10-15 female flies were raised on standard fly food and flipped every 2 days for 33 days before being put on standard fly food supplemented with 2.5 g per 100 ml of FD & C blue dye for 2 days. At 35 days each vial was scored for percentage smurf flies under a dissection microscope.

### RNA isolation, RT and quantitative PCR

For RNA isolation, 10-15 adult guts were dissected, and tissues were homogenized in TRIzol reagent (Ambion). The supernatant was collected and processed using Direct-zol RNA MicroPrep columns (Zymo Research) following the manufacturer’s protocol. Reverse transcription was performed using the iScript cDNA Synthesis Kit (Bio-Rad). Quantitative real-time PCR (qRT-PCR) was carried out on a CFX96 Real-Time System (Bio-Rad) using iQ SYBR Green Supermix (Bio-Rad). qRT-PCR reaction volume used was 10 μl (2 μl 5 μM Primer pair mix+ 5 μi 2x SYBR Green+ 3 μl cDNA). Relative mRNA levels were determined using the ΔΔCt method, with mRNA levels normalized to Rpl32.

qPCR primers:

**Table:** 

RPL32 F: 5’-AGCATACAGGCCCAAGATCG-3’			
RPL32 R: 5’-TGTTGTCGATACCCTTGGGC-3’			
ACE F 5’-AGGTGCATGTCTACACGGG-3’	PP15180	DRSC	primer
Bank			
ACE R 5’-ACGTTGGTGTTGGGGTTCC-3’	PP15180	DRSC	primer
Bank			

### Single-nuclei RNA-seq

#### Single-nucleus suspension and FACS:

Single-nucleus suspension was conducted as previously described^43^. Briefly, 70 guts per condition were dissected in cold Schneider’s medium, flash-frozen and stored at −80°C. Prior to FACs sorting, samples were spun down and Schneider’s medium was exchanged with homogenization buffer [250mM Sucrose, 10mM Tris pH8, 25mM KCl, 5mM MgCl, 0.1% Triton-X, 0.5% RNasin Plus (Promega, N2615), 50x protease inhibitor (Promega G6521), 0.1mM DTT]. Using 1ml dounce (Wheaton 357538), nuclei were released by 20 loose pestle strokes and 40 tight pestle strokes while keeping samples on ice and avoiding foam. Next, nuclei were filtered through 5 ml cell strainer (40 μm), and using 40 μm Flowmi (BelArt, H13680-0040). Nuclei were centrifuged, resuspended in PBS/0.5%BSA with 0.5% RNase inhibitor, filtered again with 40 μm Flowmi and stained with DRAQ7^™^ Dye (Invitrogen, D15106). Single nuclei were sorted with Sony SH800Z Cell Sorter at PCMM Flow Cytometry Facility at Harvard Medical School and 100k nuclei per sample were collected in PBS/BSA buffer.

#### 10x genomics and sequencing:

Single nuclei RNA-seq libraries were prepared using the Chromium Next GEM Single Cell 3’ Library and Gel Bead Kit v3.1 according to the 10xGenomics protocol. Approximately 16,500 nuclei were loaded on Chip G with an initial concentration of 700 cells/μl based on the ‘Cell Suspension Volume Calculator Table’. Sequencing was conducted with Illumina NovaSeq 6000 at Harvard Medical School Biopolymers Facility.

#### 10x data processing:

Raw sequencing data were aligned to the *Drosophila melanogaster* reference genome (BDGP6.32, Ensembl release 104) using 10x Genomics Cell Ranger v7.1.0. Low quality nuclei with less than 500 Unique Molecular Identifiers (UMI) were filtered out. The default clustering and uniform manifold approximation and projection (UMAP) generated by 10X Loupe Browser was used to cluster cells. Cell types were manually annotated using the top marker genes from each cluster. The filtered count matrix and metadata were loaded into R (v4.4.0) and further processing was performed using the default Seurat workflow (v5.2.1). Differential gene expression analysis was performed using a Mann Whitney-U test with a cut-off of p-value < 0.05 and abs(Log2FC) > .5 and percent expression > 1%.

Normalized expression violin plots and heatmaps were generated using Seurat, ggplot2 (v3.5.2), ComplexHeatmap (2.24.0) and pheatmap (v1.0.12). hdWGCNA (v0.4.05) was used to create metacells for which Pearson correlation tests were performed for candidate transcription factors and Ace expression.

### GCaMP7c live imaging

GCaMP7c live imaging was performed as previously described^24^. Guts were dissected in fresh HL3 buffer (1.5mM Ca2+, 20mM MgCl2, 5mM KCl, 70mM NaCl,10mM NaHCO3, 5mM HEPES, 115mM Sucrose, 5mM Trehalose) and placed in eight-well clear bottom cell culture chamber slides with HL3. Guts were stabilized with a Nylon mesh (Warner instruments, 64-0198) and paper clips cut in small identical pieces. ACh and Nicotine sensitivity assay: ACh and Nicotine sensitivity assay was done using LSM780 and LSM980 microscopes with 40x water objective lens. Each frame (~2.5 sec/frame) is the maximum projection of 5-6 z-stacks (2.96μm/z) and was acquired with 488nm excitation for GFP. 5mM Acetylcholine (Acetylcholine Chloride, Sigma A2661) or 1.33 mM Nicotine (Sigma, N3876) were added at the 10th frame (~25 sec) For Figure 3B-C an old aliquot of Acetylcholine was used at 10mM after testing response in WT midguts to a range of concentrations, when new aliquot was purchased 5mM concentration was used. All images were taken from similar areas in the Anterior midgut between R1-R2. Fiji was used for assembly and calculation of fluorescence per frame. DF/F0 = Ffr-F0/ F0. Ffr is the fluorescence per frame and F0 (baseline fluorescence) is the average fluorescence intensity of the first 9 frames (fr1-fr9).

### PH3+ counts

Guts were dissected and fixed in 4% PFA for 20 minutes. They were washed 3x in .1% PBST and incubated for 1 hr at RT in 5% Normal Goat Serum in .1%PBST. Guts were incubated for overnight with rabbit anti-pH3 (Millipore #06-570; 1:3000). Guts were then washed for 30 minutes 3x in .1% PBST then incubated for 2hrs at RT with secondary antibody, Alexa Fluor 555-conjugated goat anti-rabbit IgG (1:200) and DAPI (1:3000). The guts were then washed 3X in .1%PBST for 30 minutes and mounted in VECTASHIELD antifade mounting media (Vector Laboratories). Slide genotype was blinded and PH3+ nuclei were counted with an epifluorescence microscope.

### Fkh nuclear localization

Guts were dissected and fixed in 4% PFA for 20 minutes. They were washed 3x in .1% PBST and incubated for 1 hr at RT in 5% Normal Goat Serum in .1%PBST. Guts were incubated for 2 days at 4C with Rab-anti-FKH ^64^ (1:100)(gift from Dr. Chandrasekaran, St. Mary’s College of California) in 5% Normal Goat Serum in .1%PBST. Guts were then washed for 30 minutes 3x in .1% PBST then incubated overnight in secondary antibody, Alexa Fluor 555-or 647 conjugated goat anti-rabbit IgG (1:200) and DAPI (1:3000). The guts were then washed 3X in .1%PBST for 30 minutes and mounted in antifade mounting media. Fluorescence was imaged using W1 Yokogawa spinning disk, Nikon inverted Ti2 confocal microscope. Percent nuclear localization was calculated on maximum intensity projections in Fiji.

### Statistics and Reproducibility

Prism (https://www.graphpad.com/scientific-software/prism/) or ggplot2 was used to create graphs as well as to perform statistical analysis. Statistical tests are indicated in figure legends. No statistical analysis was conducted to determine sample sizes. Randomization was not performed. Blinding experiments were conducted during mitotic division counts. No data were excluded. Confocal images shown in figures are representative of two to 4 independent experiments with similar result.

## Supplementary Material

Supplement 1

2
