# Supplementary material for: Cholinergic Signaling Modulates Intestinal Pathophysiology in a Drosophila Model of Cystic Fibrosis
Source: bioRxiv. 2025 Jul 5:2025.07.02.662792. Preprint. [Version 1] doi: 10.1101/2025.07.02.662792 (PMC12236621; doi:10.1101/2025.07.02.662792)
Supplement: 2 [file NIHPP2025.07.02.662792v1-supplement-2.pdf]

bioRxiv preprint doi: https://doi.org/10.1101/2025.07.02.662792; this version posted July 5, 2025. The copyright holder for this preprint (which was not certified by peer review) is the author/funder, who has granted bioRxiv a license to display the preprint in perpetuity. It is made available under aCC-BY 4.0 International license.

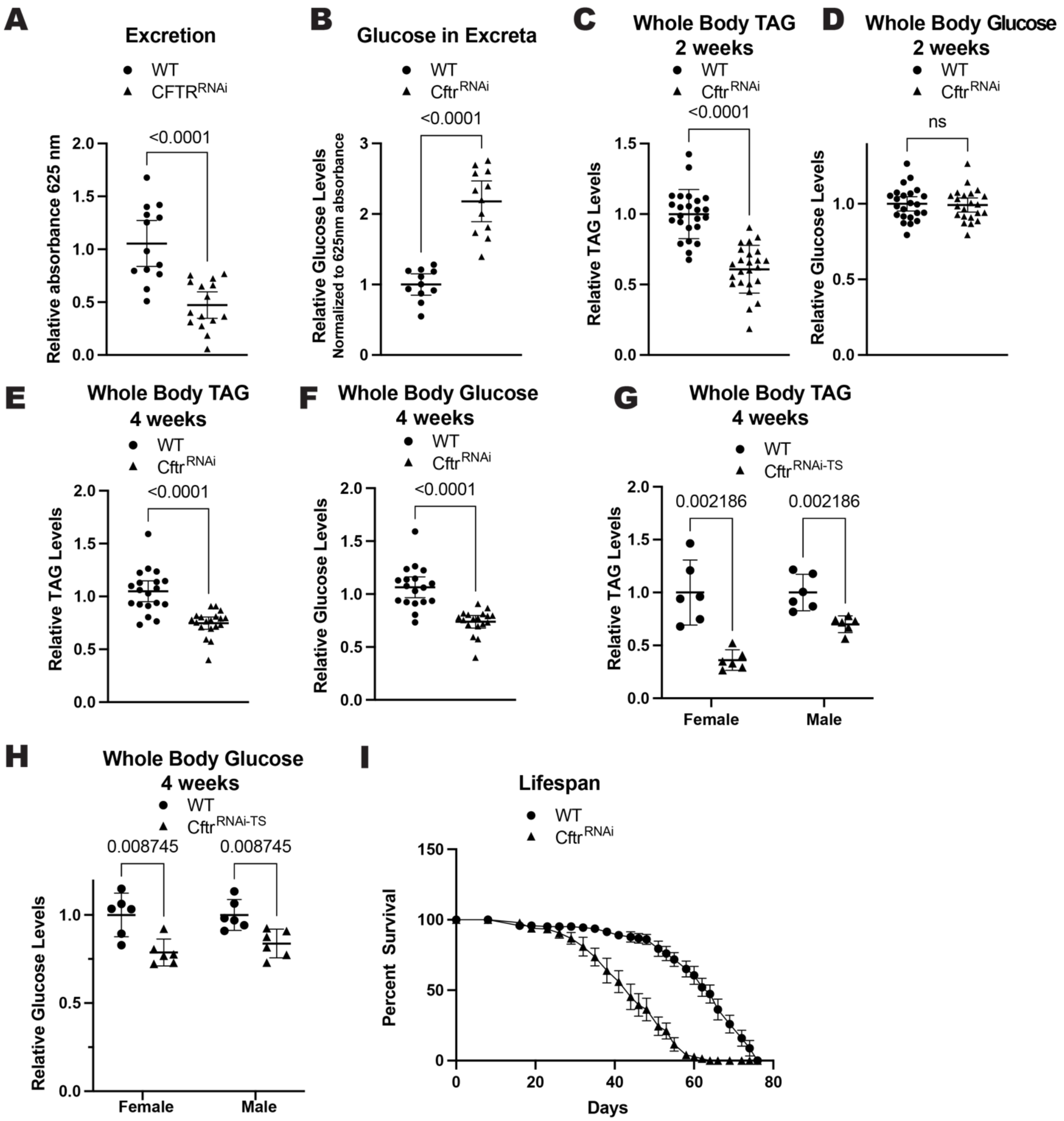

Supplementary Figure 1. Related to figure 1

(**A**) CF model guts have decreased excretion rate compared to WT guts as measured by the amount of excreta collected over a 1.75 hr time period. n= 13(WT), 15 (*Cftr^{RNAi}*) vials of 10-15 males from 3 independent crosses. **B**) CF model guts have increased glucose in excreta compared to WT flies. N= 11(WT), 12 (*Cftr^{RNAi}*) vials of 15-20 males from 2 independent crosses. (**C-F**) CF model guts have reduced whole body energy stores to WT flies. (**C**) CF model guts have reduced TAG levels at 2 weeks of age compared to WT flies. n= 24 of 8 pooled males from 4 independent crosses. (**D**) Male CF model guts have no significant difference in whole body glucose levels at 2 weeks of age compared to WT flies. n= 23 (WT), 22 (*Cftr^{RNAi}*) 8 pooled males from 4 independent crosses. (**E**) CF model gut flies have reduced whole body

bioRxiv preprint doi: https://doi.org/10.1101/2025.07.02.662792; this version posted July 5, 2025. The copyright holder for this preprint (which was not certified by peer review) is the author/funder, who has granted bioRxiv a license to display the preprint in perpetuity. It is made available under aCC-BY 4.0 International license.

TAG levels at 4 weeks of age compared to WT flies. n= 19 of 8 pooled males from 4 independent crosses. (**F**) CF model gut flies have reduced whole body glucose at 4 weeks of age compared to WT flies. n= 24 (WT), 23 (*Cftr*^{RNAi}) of 5 pooled females from 4 independent crosses. (**G-H**) Decreased whole body metabolites are not due to a developmental defect in CF model guts. (**G**) Whole body TAG levels are reduced in CF model guts compared to WT when *Cftr* knockdown is induced by temperature shift in 1-2 day old adults. n= 6. **(H)** Whole body glucose levels are reduced in CF model guts compared to WT when *Cftr* knockdown is induced by temperature shift in 1-2 day old adults. n= 6 of 5 (female) or 8 (male) pooled flies. (**A-H**) p values were calculated using the Mann-Whitney test in Graphpad prism. Error bars are mean with 95% CI. (**I**) CF model gut flies have reduced lifespan compared to WT flies. n= 11 vials with 10-15 males from 2 independent crosses. (**A-F, I**) WT is *Myo1A* > + and *Cftr*^{RNAi} is *Myo1A* > *Cftr*^{RNAi}. (**G-H**) WT is *Myo*^{TS} > + and *Cftr*$^{RNAi\text{-}TS}$ is *Myo*^{TS} > *Cftr*^{RNAi}.

bioRxiv preprint doi: https://doi.org/10.1101/2025.07.02.662792; this version posted July 5, 2025. The copyright holder for this preprint (which was not certified by peer review) is the author/funder, who has granted bioRxiv a license to display the preprint in perpetuity. It is made available under aCC-BY 4.0 International license.

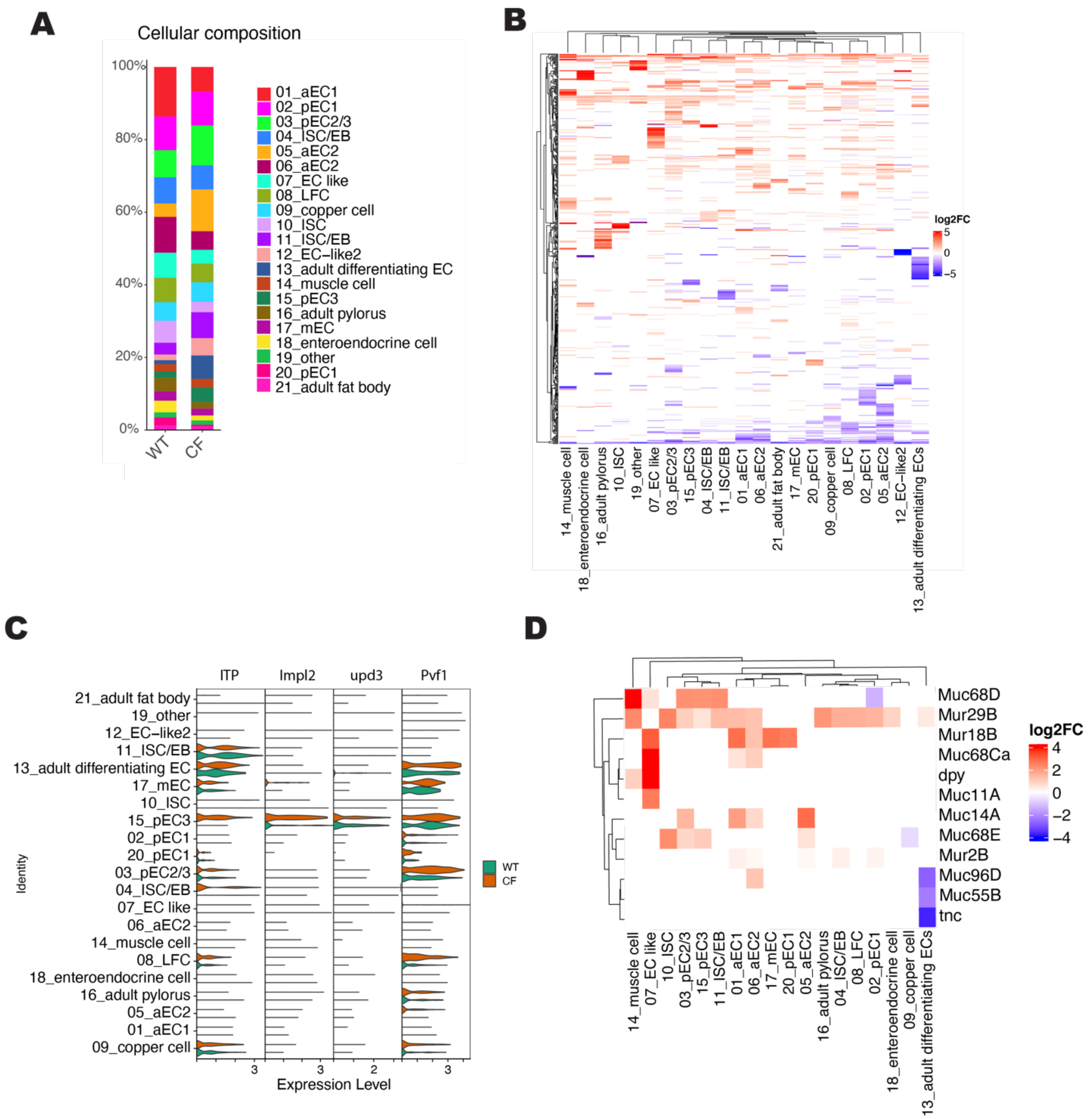

Supplementary Figure 2. Related to Figure 2

(A) Differences in cell type composition between WT and CF guts illustrated as a stacked bar chart depicting percentage of cell belonging to each cell cluster in snRNA-seq data for WT and CF model guts. **(B-C)** Many secreted peptides are differentially expressed between WT and CF guts across cell clusters. **(B)** Heat map of the differentially expressed secreted proteins. Color reflects the log2fold change of expression in CF model guts comparing to control in each cell type. **(C)** Violin plots of expression of secreted peptides important for Yki^{act} gut tumor physiology (*Itp, Impl2, upd3,* and *pvf1*) in snRNA-seq data. **(D)** Heat map of log2 fold change of mucin gene expression in each cell cluster indicates mucin gene expression is over all upregulated in CF model gut cells compared to WT.

bioRxiv preprint doi: https://doi.org/10.1101/2025.07.02.662792; this version posted July 5, 2025. The copyright holder for this preprint (which was not certified by peer review) is the author/funder, who has granted bioRxiv a license to display the preprint in perpetuity. It is made available under aCC-BY 4.0 International license.

bioRxiv preprint doi: https://doi.org/10.1101/2025.07.02.662792; this version posted July 5, 2025. The copyright holder for this preprint (which was not certified by peer review) is the author/funder, who has granted bioRxiv a license to display the preprint in perpetuity. It is made available under aCC-BY 4.0 International license.

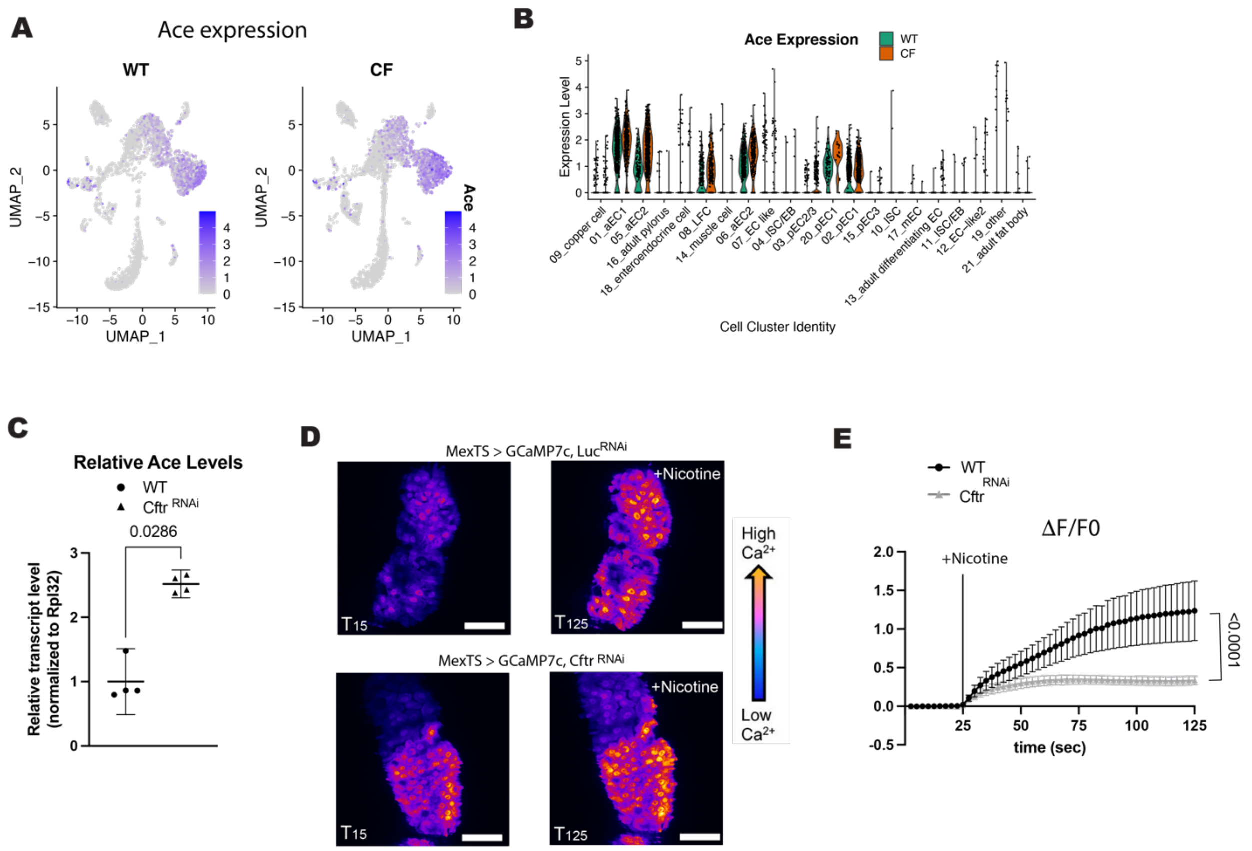

Supplementary Figure 3. Related to Figure 3

(A-B) Ace expression is upregulated *Cftr* deficient midguts compared to WT midguts in snRNA-seq data. **(A)**Ace expression in each cell in WT and *Cftr* deficient guts plotted onto UMAP **(B)** Violin plots of Ace expression in each cell cluster identified in snRNA-Seq data set. **(C)** *Ace* expression is increased in *Cftr* deficient guts compared to WT guts via qPCR analysis of whole guts. n=4 of 10-15 pooled guts from 1 independent experiment. **(D)** Representative images of GCaMP7c fluorescence, in WT (*MexTS* > *GCaMP7c, LucRNAi*) and *Cftr* deficient guts (*MexTS* > *GCaMP7c, Cftr^{RNAi}*) before (T15s) or after addition of Nicotine (T125). Scale bars are 50 µm. **(E)** Graph of average relative fluorescent intensity, ΔF/F0, per frame (2.5s per frame) and genotype. n= 8 (WT) and 7 (*Cftr^{RNAi}*) from 3 independent experiments. Error bars are mean +/- SEM and pValue was calculated using the Mann-Whitney test in Graphpad prism.

bioRxiv preprint doi: https://doi.org/10.1101/2025.07.02.662792; this version posted July 5, 2025. The copyright holder for this preprint (which was not certified by peer review) is the author/funder, who has granted bioRxiv a license to display the preprint in perpetuity. It is made available under aCC-BY 4.0 International license.

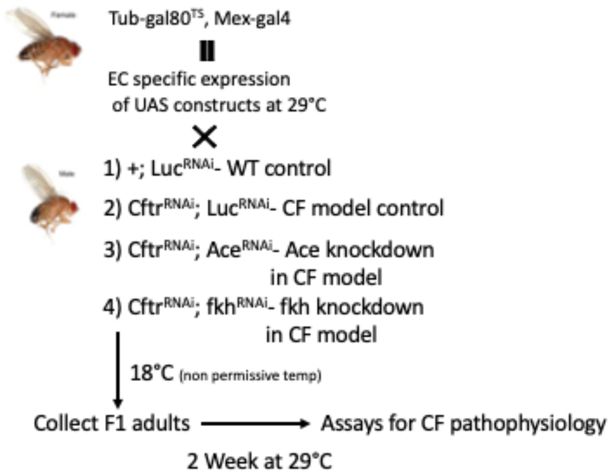

Supplementary Figure 4. Related to Figure 3-5
Experimental set up for figures 3D-E, 4, and 5D-H. *MexTS* (*Tub-gal80^{TS}, Mex-gal4*) virgin females were crossed to males with the indicated UAS-RNAi constructs and raised at 18C, nonpermissive temperatures. 1-3 day old adults were moved to 29C for 2 weeks (35 days for smurf assay) and were than used in indicated assays.

bioRxiv preprint doi: https://doi.org/10.1101/2025.07.02.662792; this version posted July 5, 2025. The copyright holder for this preprint (which was not certified by peer review) is the author/funder, who has granted bioRxiv a license to display the preprint in perpetuity. It is made available under aCC-BY 4.0 International license.

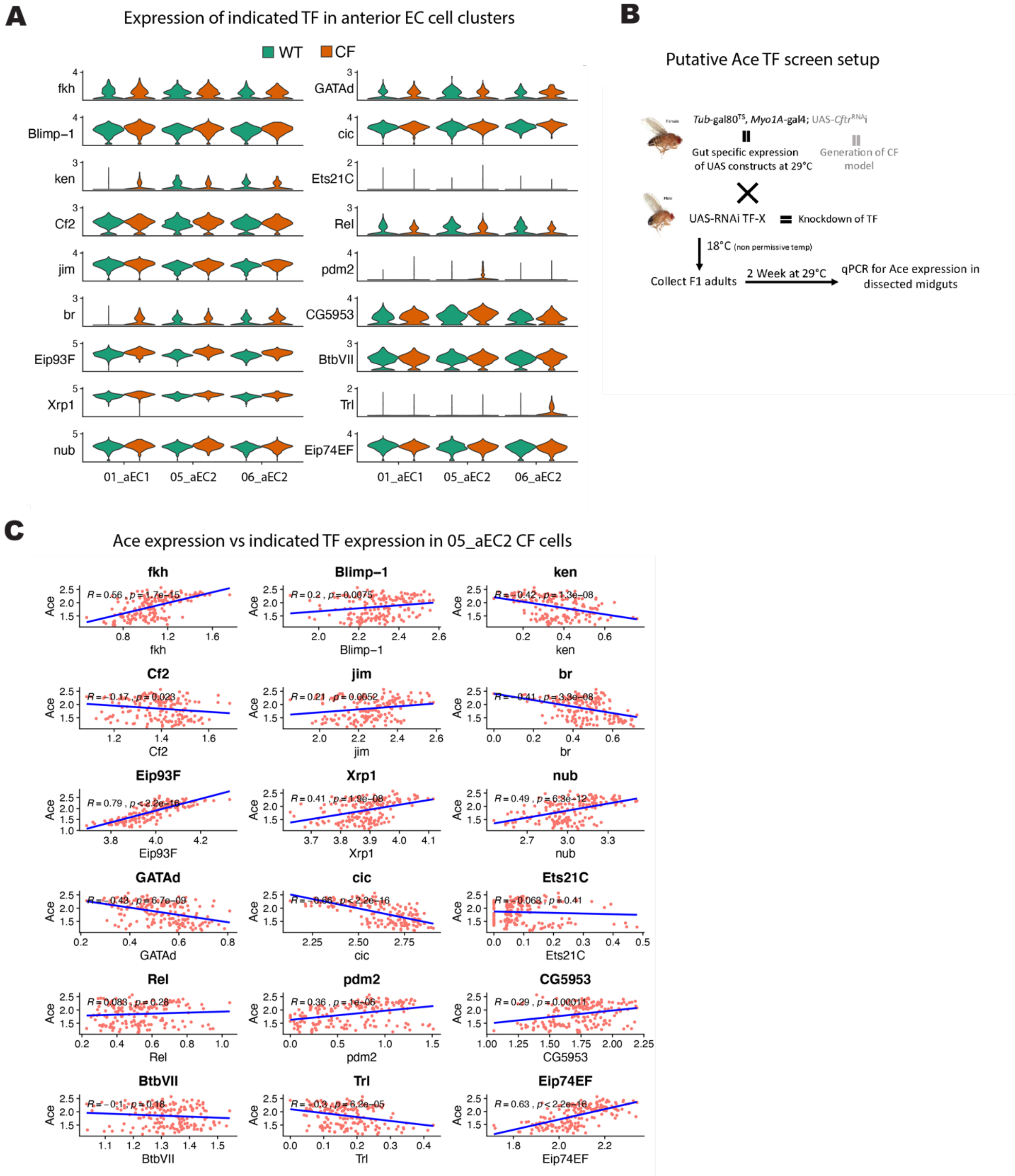

Supplementary Figure 5. Related to Figure 5

(A) Violin plots of expression of candidate *Ace* transcription factors in anterior EC cell clusters from snRNA-seq data. **(B)** Diagram of experimental set up for *Ace* transcription factor screen. *MyoTS* (*Tub-gal80TS, Myo1A-gal4*); *UAS-Cftr*^{RNAi} females were crossed to male flies with UAS-RNAi against candidate transcription factor and raised at 18C, nonpermissive temperatures. 1-3 day old adults were moved to 29C for 2 weeks and guts were dissected to assay for *Ace* expression levels. **(C)** Scatter plots showing correlation of *Ace* and candidate transcription factor

bioRxiv preprint doi: https://doi.org/10.1101/2025.07.02.662792; this version posted July 5, 2025. The copyright holder for this preprint (which was not certified by peer review) is the author/funder, who has granted bioRxiv a license to display the preprint in perpetuity. It is made available under aCC-BY 4.0 International license.

expression levels in meta-cells from the 05-aEC2 CF cell cluster (Cell cluster with overall highest *Ace* expression).

bioRxiv preprint doi: https://doi.org/10.1101/2025.07.02.662792; this version posted July 5, 2025. The copyright holder for this preprint (which was not certified by peer review) is the author/funder, who has granted bioRxiv a license to display the preprint in perpetuity. It is made available under aCC-BY 4.0 International license.

TF2TG							
TF FBgn	TF Symbol	Peak Count	Motif Count	REDfly TFBS Count	Location	Protein-Protein Interactors	Genetic Interactors
FBgn0000659	fkh	1	2	0	intragenic	Ada2b	
FBgn0000659	fkh	1	2	0	upstream	Ada2b	
FBgn0035625	Blimp-1	2	5	0	upstream		
FBgn0035625	Blimp-1	0	7	0	intragenic		
FBgn0011236	ken	0	1	0	intragenic	pzg, Ada2b, E(bx), Trl	
FBgn0011236	ken	0	1	0	upstream	pzg, Ada2b, E(bx), Trl	
FBgn0000286	Cf2	0	1	0	intragenic	bin	
FBgn0027339	jim	0	20	0	intragenic		
FBgn0027339	jim	0	1	0	upstream		
FBgn0283451	br	1	0	0	upstream	Ada2b, Rel, rib	Met (pubmed)
FBgn0283451	br	0	1	0	intragenic	Ada2b, Rel, rib	Met (pubmed)
FBgn0264490	Eip93F	0	1	0	intragenic		
FBgn0261113	Xrp1	1	0	0	upstream	Irbp18	
FBgn0261113	Xrp1	0	2	0	intragenic	Irbp18	
FBgn0085424	nub	0	5	0	intragenic	pdm2, wek	
FBgn0085424	nub	0	4	0	upstream	pdm2, wek	
FBgn0032223	GATAd	0	5	0	upstream	mam, Ada2b	
FBgn0262582	cic	2	0	0	upstream	gro	Dl (pubmed)
FBgn0262582	cic	0	1	0	intragenic	gro	Dl (pubmed)
FBgn0005660	Ets21C	0	1	0	upstream		
FBgn0014018	Rel	2	1	0	upstream	htk, Dif, dl, pzg, br, grh, sqz	
FBgn0004394	pdm2	0	3	0	intragenic	salr, nub	
FBgn0004394	pdm2	0	4	0	upstream	salr, nub	
FBgn0032587	CG5953	0	1	0	upstream	knrl	
FBgn0263108	BtbVII	0	1	0	intragenic	CG32121	
FBgn0013263	Trl	3	0	0	intragenic	CG12155, CG8924, psq, Ssrp, ken, pzg, E2f1, bab2, lola, E(bx), Gug, Adf1, ttk	
FBgn0013263	Trl	2	0	0	upstream	CG12155, CG8924, psq, Ssrp, ken, pzg, E2f1, bab2, lola, E(bx), Gug, Adf1, ttk	
FBgn0264490	Eip93F	0	1	0	intragenic		

Supplementary Table 1: Related to Figure 5

TF2TG results for TFs with ChIP-seq Peak counts and/or binding motifs within 5 kb of *Ace* gene that were included in the transcription factor screen for *Ace* transcription (Fig 5A).
